# Effects of three spontaneous ventilation modes on respiratory drive and muscle effort in COVID-19 pneumonia patients

**DOI:** 10.1186/s12890-023-02631-0

**Published:** 2023-09-08

**Authors:** José Manuel Serrano Simón, Carolina Joya Montosa, Juan Francisco Martínez Carmona, Manuel Jesús Delgado Amaya, Javier Luna Castro, Ashlen Rodríguez Carmona, José Castaño Pérez, Marina Rodríguez Delgado, Guillermo Besso Centeno, José Antonio Benítez Lozano

**Affiliations:** 1 Intensive Care Service, Hospital Universitario Reina Sofía, Córdoba, Spain; 2 Intensive Care Service, Hospital La Merced, Osuna, Seville Spain; 3 Intensive Care Service, Hospital Regional Universitario de Málaga, Málaga, Spain; 4 Unidad Terapia Intensiva, Hospital El Carmen, Mendoza, Argentina; 5 Intensive Care Service, Hospital Virgen de las Nieves, Granada, Spain; 6 Unidad Terapia Intensiva, Clínica del Aconcagua, Villa Mercedes, San Luis Argentina

**Keywords:** COVID-19 pneumonia, Muscle pressure, Respiratory drive, Muscle effort, Respiratory muscle monitoring, Mechanical ventilation, Respiratory mechanics, Patient self-inflicted lung injury (P-SILI)

## Abstract

**Background:**

High drive and high effort during spontaneous breathing can generate patient self-inflicted lung injury (P-SILI) due to uncontrolled high transpulmonary and transvascular pressures, with deterioration of respiratory failure. P-SILI has been demonstrated in experimental studies and supported in recent computational models. Different treatment strategies have been proposed according to the phenotype of elastance of the respiratory system (Ers) for patients with COVID-19. This study aimed to investigate the effect of three spontaneous ventilation modes on respiratory drive and muscle effort in clinical practice and their relationship with different phenotypes. This was achieved by obtaining the following respiratory signals: airway pressure (Paw), flow (V´) and volume (V) and calculating muscle pressure (Pmus).

**Methods:**

A physiologic observational study of a series of cases in a university medical-surgical ICU involving 11 mechanically ventilated patients with COVID-19 pneumonia at the initiation of spontaneous breathing was conducted. Three spontaneous ventilation modes were evaluated in each of the patients: pressure support ventilation (PSV), airway pressure release ventilation (APRV), and BiLevel positive airway pressure ventilation (BIPAP). Pmus was calculated through the equation of motion. For this purpose, we acquired the signals of Paw, V´ and V directly from the data transmission protocol of the ventilator (Dräger). The main physiological measurements were calculation of the respiratory drive (P0.1), muscle effort through the ΔPmus, pressure‒time product (PTP/min) and work of breathing of the patient in joules multiplied by respiratory frequency (WOBp, J/min).

**Results:**

Ten mechanically ventilated patients with COVID-19 pneumonia at the initiation of spontaneous breathing were evaluated. Our results showed similar high drive and muscle effort in each of the spontaneous ventilatory modes tested, without significant differences between them: median (IQR): P0.1 6.28 (4.92–7.44) cm H_2_O, ∆Pmus 13.48 (11.09–17.81) cm H_2_O, PTP 166.29 (124.02–253.33) cm H_2_O*sec/min, and WOBp 12.76 (7.46–18.04) J/min. High drive and effort were found in patients even with low Ers. There was a significant relationship between respiratory drive and WOBp and Ers, though the coefficient of variation widely varied.

**Conclusions:**

In our study, none of the spontaneous ventilatory methods tested succeeded in reducing high respiratory drive or muscle effort, regardless of the Ers, with subsequent risk of P-SILI.

## Background

An increase in respiratory drive has been described for COVID-19 patients with moderate or severe respiratory failure [1]. The strong efforts of these patients are noteworthy and compatible with the development of potential patient self-inflicted lung injury (P-SILI) due to uncontrolled transpulmonary and transvascular pressures [2], such as deterioration and relapses of respiratory failure [1]. To induce hyperventilation in experimental settings, Mascheroni et al. [3] infused salicylate into the brainstem of spontaneously breathing sheep. The increase in minute ventilation produced lung injury, which was prevented by sedation and paralysis on controlled mechanical ventilation. In addition, two patterns of Ers have been described in such patients: high and low Ers [4]. Based on the P-SILI hypothesis and Ers, different authors have advocated for radical changes in respiratory management [4, 5]. However, the respiratory mechanics just before switching to spontaneous breathing or at the time of estimation of the respiratory drive, as well as the relationship between both, have not been sufficiently studied. P-SILI in COVID-19 has been evaluated in a computational model, which demonstrated that inspiratory effort is comparable with that associated with ventilator-induced injury [6].

Identifying strategies that can mitigate progression of lung injury is of interest for these patients. The aim of the present work was to evaluate the impact of different spontaneous ventilation modes on the respiratory response regarding respiratory drive, muscle effort, and WOBp in mechanically ventilated COVID-19 patients via direct acquisition of monitored respiratory signals from ventilators: Paw, V´ and V. The goal was to evaluate possible reduction in muscle effort, as well as its relationship with the Ers of the respiratory system (Ers). This study focused on inspiratory muscle activity by calculating muscle pressure (Pmus); we thus avoided more invasive manoeuvres with a risk of contamination and contagion, such as oesophageal catheters. Some of the results of this study have been previously reported in abstract form [7].

## Methods

### Design of the study

The present study was conceived as a case series of physiological analyses from a single centre in the medical-surgical intensive care unit (ICU). The study was included in the original project on muscle pressure approved by the local Ethics Committee of the Hospital Reina Sofía Córdoba, with reference PM-12. All patients and/or their surrogates were informed, and written consent was given prior to inclusion in the study.

### Study population

We considered eligible for inclusion all consecutive patients older than 18 years admitted to the ICU with a confirmed diagnosis of COVID-19 (by real-time PCR) who were on assisted mechanical ventilation with pressure support ventilation (PSV) or suitable for PSV as per clinical decision. All patients were ventilated with an Evita 2D or XL ventilator (Dräger Medical, Lübeck, Germany). Sedation was titrated to achieve a level on the Richmond Agitation and Sedation Scale (RASS) of -2 to -3. All patients were admitted to the ICU from the hospitalization ward.

### Study procedures

#### Data acquisition

We collected demographic data (age, sex, body mass index [BMI]), comorbidities, severity scores (APACHE II) upon ICU admission, chronology (time on ICU admission and mechanical ventilation), vital signs, laboratory parameters, and outcome.

#### Respiratory signals

Paw, V’ and V were recorded using the Medibus® protocol of the Evita 2D or XL® Dräger ventilator at a sampling rate of 125 Hz using personal software.

After clinical stabilization under assist-volume control mode (ACV), three spontaneous breathing trials were performed in random order for all patients. Ventilator settings during spontaneous breathing were as follows:

- PSV 10 (5–15) cm H_2_O with PEEP 10 cm H_2_O.
- APRV High pressure 20 cm H_2_O, time at inspiration for ≅ 90% of each respiratory cycle and the time at expiration set to finish at 75% of the peak expiratory flow; thus, the time low was sufficiently brief to maintain PEEP.
- BiLevel (BIPAP®) 20 on 10 cm H_2_O, inspiratory/expiratory time ≅ 2.5/1.

In this way, the delta pressure (ΔP) was maintained at a safety threshold < 15 cm H_2_O [8]. Spontaneous ventilation modes were stopped if any sign of poor tolerance occurred, such as tachypnoea > 35 respiratory rate (RR), clinical signs of muscle fatigue, altered level of consciousness or SpO2 < 92%. Between each test, the patient was ventilated in the ACV for 20–30 min to achieve a cardiorespiratory status equivalent to baseline. The time period of recording was 60 min in each ventilatory mode.

#### Physiological measurements

The three components of the equation of motion (EM) for the respiratory system, elastance of the respiratory system (Ers), total resistances (Rrs) and total PEEP (PEEPt), were obtained in the passive mode using multiple linear regressions between Paw, V´ and V by the least square fitting method. In addition, in each of the spontaneous modes tested, Ers and Rrs were obtained for accurate calculation of Pmus. In spontaneous modes, we use the inspiratory and expiratory occlusion manoeuvres for Ers and measurement of the time constant (t), as the time required for 25–75% of the exhaled volume, to obtain Rrs [9]. PEEPt during spontaneous breathing modes was obtained by the end-inspiration occlusion method through the formula Pplat, aw = Elastic pressure (Pel) + PEEPt [10], Pel = V * Ers. In this way, the signals of Pmus and total distending pressure were obtained according to the formula PDist_EM = Ers*V + V’*Rrs + PEEPt. Pmus = Paw – PDist_EM. The mean PDist_EM was assumed to be an estimate of the total lung and chest wall stress. According to the standard elastance of the chest wall (Ecw), the main component of the distending pressure corresponds to the total lung stress.

Parameters related to the respiratory drive (P0.1), effort (ΔPmus, PTP/min), work of breathing (WOBp) as joules/min, and breathing pattern were calculated in each ventilatory mode, cycle by cycle, as computed according to the following formulas:

- P0.1 = ∆Poccl cm H_2_O (100 ms),
- $$\mathrm{PTP}/\mathrm{min}= \left({\int }_{0}^{\mathrm{Ti}}\mathrm{Pmus dt}\right)*\mathrm{RR}$$,
- $$\mathrm{WOBp}={\int }_{0}^{\mathrm{Ti}}\left(\mathrm{Pmus}\left(\mathrm{t}\right)*\mathrm{V^{\prime}}(\mathrm{t})\right)\mathrm{dt}$$.

The inspiratory period was obtained through the flow signal from the onset of the inspiratory flow to the cycling off at flow zero.

Measurements were repeated at least three times for the occlusion manoeuvre, inspiratory and expiratory, separated by at least 1 min, and the average values were recorded. The analysed period corresponded to an average of 500 ± 23 breath cycles in each mode and patient.

#### Statistical analysis

Continuous variables are expressed as the median and interquartile range (IQR). Data were compared by one-way repeated measure ANOVA with the rank sum test because of the small sample size. Linear regression was used to assess the relationship between respiratory drive and WOBp with respect to Ers. Basic calculations were performed in Excel v.2019. Sigmaplot v.14 was used for statistical analysis.

## Results

### Patients

Eleven mechanically ventilated patients with COVID-19 pneumonia admitted to the ICU over a 2-month period (April–May 2020), corresponding to the first wave in Spain, were considered for inclusion. One patient was excluded because he was on treatment with venovenous extracorporeal membrane oxygenation (VV-ECMO). A total of 10 patients were studied. A physiological study comparing the three ventilation modes was performed in each of the patients studied. The study protocol was successfully applied to all patients. The characteristics of the patients are displayed in Table 1. They had been in the clinical ward for a median of 4 (2–7) days, and they had received respiratory treatment with high nasal flow and/or noninvasive ventilation.

**Table 1:** Baseline characteristics of the studied patients

Patient (N)	Sex	Age (yr)	Apache II score	BMI (Kg/m^2^)	Comorbidities	Days on ventilator	pH	PaO_2_/FiO_2_ (mmHg)	FiO_2_	PCO_2_ (mmHg)	Lactic mmol/L
1	M	53	20	35	Kidney transplantation	10	7.48	198	0.4	35	0.60
2	M	57	18	32	Hypertension	10	7.14	193	0.4	70	1.72
3	F	76	17	26	Hypertension	5	7.25	273	0.4	62	1.10
4	M	60	15	28	Hypertension	24	7.25	185	0.4	54	0.70
5	F	66	19	35	Diabetes	22	7.39	167	0.6	63	1.81
6	M	52	21	30	Lymphoma	6	7.33	193	0.4	40	1.43
7	F	76	18	27	Hypertension	5	7.43	154	0.5	54	0.90
8	M	47	20	30	COPD	26	7.27	164	0.5	32	0.54
9	M	61	19	27	Abdominal surgery	14	7.43	266	0.35	47	1.21
10	F	69	21	28	Abdominal surgery	19	7.26	156	0.45	60	1.21
Median (IQR)		61 (53–71)	19 (18–20)	29 (27–33)		12 (6–23)	7.30 (7.25–7.43)	189 (162–267)	0.40 (0.40–0.50)	54 (39–62)	1.15 (0.67–1.50)

Results are presented as median and interquartile range (IRQ)

*F* Female, *M* Male, *BMI* body mass index, *COPD* Chronic obstructive pulmonary disease, *FiO*_*2*_ Fractional inspired oxygen tension

### Respiratory parameters, respiratory drive and inspiratory muscle efforts

The results of the respiratory parameters of the applied ventilation modes are given in Table 2. There were no significant differences between the various strategy trials in terms of parameters related to respiratory drive (P0.1) and inspiratory effort (∆Pmus, PTP/min or WOBp J/L). It is particularly notable that the value of P0.1, as representative of the drive, was > 5 cm H_2_O in 7 (70%) patients, with a median > 6 cm H_2_O in each of the ventilatory trials. Additionally, the median ∆Pmus was > -14 cmH_2_O, and the WOBp was > 12 J/min. The median PDist_EM (mean pressure) was > 16 cm H_2_O. These parameters suggest high respiratory drive, inspiratory effort, and WOBp, which indicate lung stress.

**Table 2:** Comparison of respiratory parameters between the applied ventilation modes

	ACV	PSV 10 (5–15 cmH_2_O)	APRV	BIPAP (20/10 cmH_2_O)	*P*
Respiratory frequency (breaths/min)	24.12 (23.31–26.42)	19.26 (16.74–21.61)	19.59 (12.14–29.35)	20.11 (13.53–24.94)	0.998
Tidal volume (ml/Kg)	4.37 (4.02–5.95)	7.65 (4.49–9.28)	5.86 (3.78–6.03)	4.58 (3.77–7.08)	0.131
pH (units)	7.33 (7.25–7.43)	7.35 (7.31–7.44)	7.33 (7.26–7.41)	7.32 (7.26–7.38)	0.965
PCO_2_ (mmHg)	54 (44–62)	43 (24–55)	57 (42–65)	53 (43–67)	0.721
PO_2_ (mmHg)	81 (77–95)	80 (63–112)	74 (64–103)	79 (68–105)	0.651
FiO_2_ (%)	40 (40–50)	40 (40–50)	40 (40–50)	40(40–50)	N/A
Total PEEP (cmH2O)	11.05 (10.31–11.55)	10.05 (8.42–10.50)	10.00 (4.45–10.81)	10.04 (9.51–10.67)	N/A
Ers (cmH_2_O/L)	25.98 (20.17–37.78)	22.24 (20.09–29.19)	25.69 (18.96–29.50)	24.75 (14.75–29.68)	0.688
Rrs (cmH_2_O/L/s)	12.15 (10.05–14.61)	15.10 (11.50–18.58)	14.76 (11.99–20.85)	11.84 (10.06–12.67)	0,117
P0.1 (cmH_2_O)	N/A	6.12 (3.69–8.26)	5.82 (4.13–10.89)	5.80 (4.22–8.85)	0.907
Delta Muscle Pressure (cmH_2_O)	N/A	13.04 (10.06–17.35)	15.77 (10.85–23.38)	16.16 (6.92–17.76)	0.556
PTPpmus (cmH_2_O.s/min)	N/A	135.17 (96.55–175.27)	148.74 (112.38–259.94)	156.95 (84.18–335.11)	0.716
WOB (Joule/min)	N/A	13.52 (5.34–18.97)	10.54 (4.87–24.75)	14.39 (4.10–23.78)	0.973
PDist_EM (cmH_2_O)	N/A	14.81 (13.97–16.03)*	20.74 (19.75–22.39)	18.91 (17.72–23.65)	< 0.001

*ACV* Assist-control volume ventilation, *PSV* Pressure support ventilation (Inspiratory pressure), *APRV* Airway pressure release ventilation (High pressure 20 cmH_2_O, expiration limited to 75% expiratory flow), *BIPAP* biLevel mode, *PEEP* Positive end-expiratory pressure, *Ers* Elastance respiratory system, *Rrs* Resistance respiratory system, *P0.1* Airway occlusion pressure generated at the airways during the first 100 ms of an inspiratory effort, P*TPpmus* Muscle pressure time product during the inspiratory period, *WOB* Patient work of breathing, *PDist_EM* Total distending pressure by equation motion, *N/A* Not applicable

^*^p < 0.05 between PSV versus APRV or BIPAP

There were no significant differences in ventilation among the ventilatory modes applied; however, there was a trend towards better PCO_2_ control during PSV (46 mmHg) vs. APRV (56 mmHg) and BiPAP (53 mmHg). Therefore, PSV might be better tolerated by the patients. We found significant differences in relation to mean PDist_EM in favour of PSV (14.81 cmH_2_O) vs. APRV (20.74 cm H_2_O) and BIPAP (18.91 cm H_2_O) (*p* < 0.001), which might be explained mainly by an extended time of inspiration (Table 2).

**Fig. 1:**
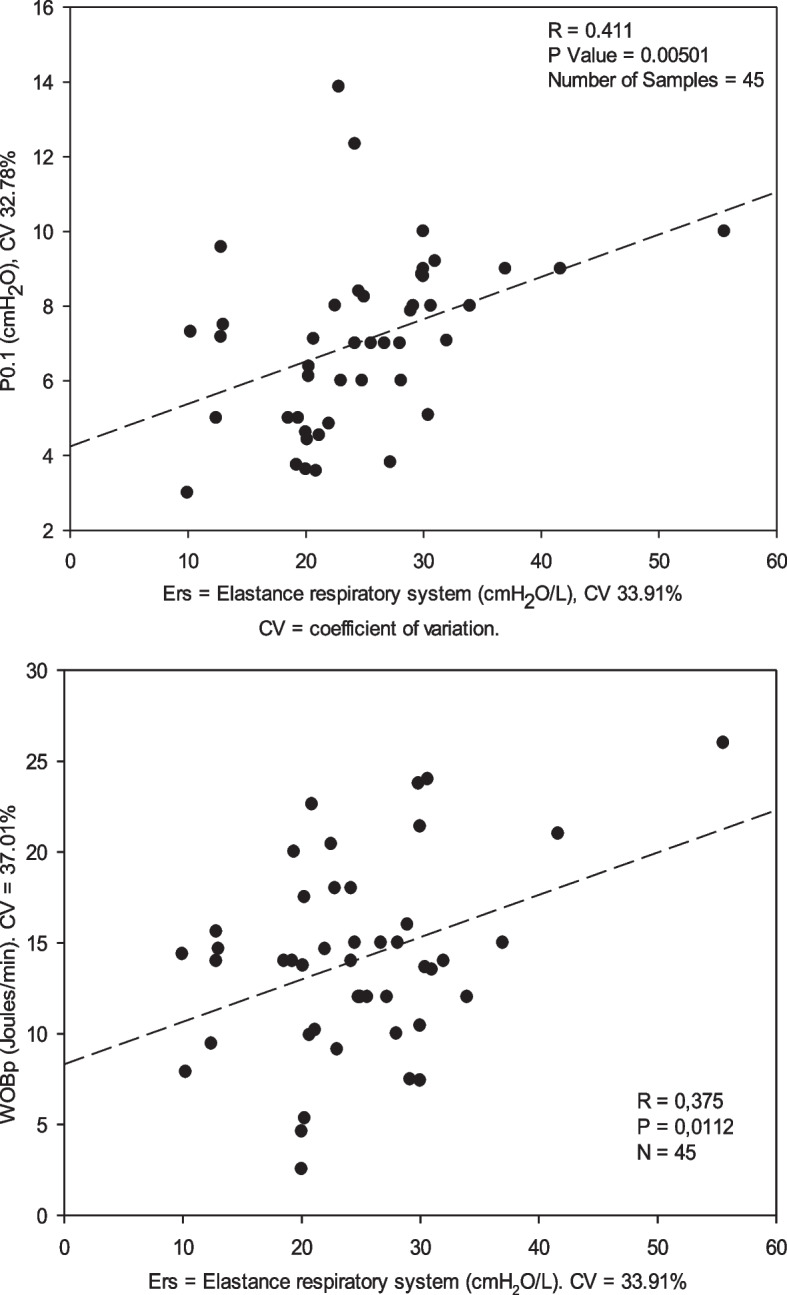
Scatter plot of Linear regression analysis for all data during the spontaneous modes to evaluate the relationship of the respiratory driver (P0.1, cmH_2_O) and work of breathing patient (WOBp, J/min), with respect to the Elastance respiratory system (Ers). Although there is a significant relationship between the respiratory driver and WOBp with respect to Ers, the increase in respiratory drive and work is only partially explained by the increase in Ers, 41% in the case of the respiratory drive and 37.5% for work

The relationships between respiratory drive, WOBp and Ers are shown in Fig. 1. Linear regression indicates a positive significant trend for both respiratory drive and work with respect to Ers. Thus, 41% of the increase in respiratory drive would be explained by the increase in the Ers and a 37.5% increase in the WOBp.

**Fig. 2:**
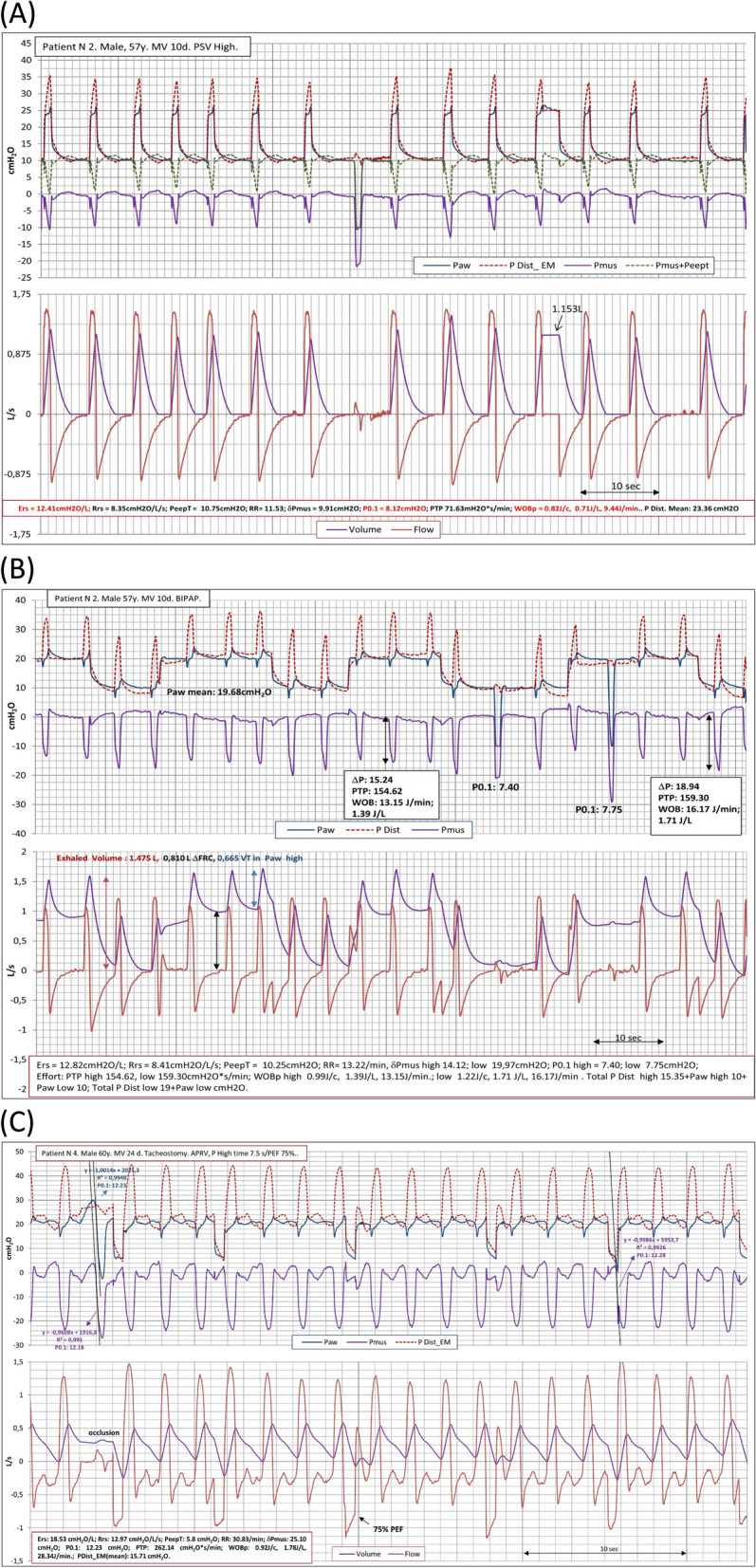
Recording of airway pressure (Paw), muscle pressure (Pmus), distending pressure by equation motion (PDist_EM), Flow, and Volume of representative patients during the ventilatory strategies applied. **A:** Patient 2, High level of PSV. **B:** BIPAP, where the effort and work is higher at lower pressure level. **C:** Patient 4, APRV. Note that respiratory drive and muscle effort parameters, are higher in all ventilatory modes, regardless the elatance of respiratory system (Ers)

Table 3 shows the individual values of the respiratory components, ICU stay, and survival of the patients studied. Figure 2 shows the graphs of a representative patient with low Ers during the three ventilatory strategies tested. We found high respiratory drive, inspiratory effort, and WOBp, even with high assistance in PSV mode. The results indicate poor prognosis.

**Table 3:** Average of the individual values of the respiratory parameters over to the three different breathing modes and hospital outcome

Patient	Ers cmH_2_O/L	Rrs cmH_2_O/L/s	P0.1 cmH_2_O	Delta Pmus cmH_2_O	PTPpmus cmH_2_O*s/min	WOB joules/min	Distending pressure cmH_2_O	ICU stay days	Survival
1	10.09	9.47	5.87	12.03	184.13	11.12	20.71	32	Yes
2	12.41	8.41	8.12	16.46	107.85	13.99	15.86	15	No
3	24.28	23.14	3.66	11.08	166.29	5.91	14.39	6	Yes
4	20.25	12.97	6.12	22.08	176.38	17.51	15.78	154	Yes
5	48.61	12.15	7.21	17.81	260.43	19.63	17.52	60	No
6	22.48	11.34	6.43	8.84	130.24	11,87	18.96	8	No
7	30.65	18.56	5.08	12.53	253.33	13.65	14.92	96	Yes
8	24.19	13.11	12.34	32.36	335.11	29.77	18.52	54	No
9	20.27	11.67	4.43	15.24	124.02	7.47	20.29	99	No
10	30.01	16.01	7.07	13.48	132.17	7.41	16.44	43	Yes
Median (IQR)	22.74 (18.29–30.16)	12.56 (10.87–16.64)	6.28 (4.92–7.44)	14.36 (11.91–18.88)	180.26 (128.51–255.11)	12.76 (7.46–18.04)	16.97 (15.57–19.29)	48.50 (13.25–96.75)	5 (50%)

*Ers* Elastance respiratory system, *Rrs* Resistance respiratory system, *P0.1* Airway occlusion pressure generated at the airways during the first 100 ms of an inspiratory effort, *PTPpmus* Muscle pressure time product during inspiratory period, *WOB* Patient work of breathing

## Discussion

The present study suggests that patients with pneumonia due to COVID-19 present an increase in respiratory drive and muscular effort compared to conventional values for patients under assisted mechanical ventilation [11, 12]. These results were obtained irrespective of any of the three ventilatory strategies applied. In our study, the ventilation modes chosen were based on protective strategies of assisted pressure in biphasic modes and maintaining ΔP below a safety threshold of 15 cm H_2_O [8]. We chose PSV as usual during spontaneous ventilation and two ventilation modes based on CPAP: APRV with a short exhalation period, similar to that proposed by Nieman et al. [13], a time-controlled adaptive ventilation protocol, and ventilation BiLevel (BIPAP) with extended inspiratory time.

The keystone for our study is obtaining the Pmus signal generated from flow, volume, and airway pressure using EM [14] by calculating Ers and Rrs both in ACV as in each spontaneous mode, and we did not find significant differences between the modes applied. Gattinoni and colleagues [4] reported patients with phenotypes with high and low compliance of the respiratory system, even on the days of mechanical ventilation on which they were included in the study. The accuracy of the Pmus calculation depends on the accuracy of the Ers and Rrs calculations, which did not show significant differences according to the applied ventilation mode. Therefore, we consider that Pmus is reliable.

The main finding of this study is that our patients had a high respiratory drive, P0.1 > 6 cm H_2_O; inspiratory effort, ΔPmus -12 to -19 cm H_2_O, PTP/min > 250 cm H_2_O.sec/min in the 3^rd^ percentile; and WOBp > 12 J/min in all the ventilatory modes tested. High respiratory drive and inspiratory effort occur in both high and low elastance.

In the context of ARDS caused by COVID-19, a recent study by Esnault and colleagues [1] demonstrated an association between increased respiratory drive and worsening respiratory function during weaning from MV, which might be a consequence of P-SILI, myotrauma (diaphragm injury) and nonresolution of COVID-19 pneumonia. Therefore, it is important to monitor respiratory parameters related to drive and inspiratory effort. This study was also performed in patients in APRV and PSV modes.

The high drive might be caused by hypoxaemia and/or hypercapnia; however, high drive by itself has been shown in COVID-19 patients [1], as confirmed by our results. None of the patients studied had increased temperature or elevated lactic acid as sepsis markers (Table 1). The relationship between respiratory drive and WOBp with respect to Ers (Fig. 1) is significant; however, the increase in respiratory drive associated with Ers is explained in 41% of cases and associated with WOBp in only 37.5% of cases, with a wide coefficient of variation, which suggests the existence of other factors involved in addition to Ers. High respiratory drive and inspiratory effort can generate an increase in volume, especially in PSV mode (hidden volume), particularly in patients with low Ers. High volume per se can cause lung injury by strain, similar to ARDS of other aetiologies due to the relationship of volume to the functional residual capacity (FRC) [15]. Large tidal volume and transpulmonary pressure increase lung oedema. This is a manifestation of P-SILI, with worsening respiratory failure and a delay in the weaning process of MV, and it can affect length of stay [1].

The ΔPmus measurements were > -12 to -16 cm H_2_O (median) for the three spontaneous breathing tests, indicating excessive respiratory efforts. Indeed, Bertoni and colleagues suggest as a potential target that Pmus values similar to those of healthy subjects breathing at rest may be safe and may prevent diaphragm atrophy, a range between 5 and 10 cm H_2_O [5].

The normal value of WOBp expressed in joules/min in a healthy subject is approximately 2.4–7.5 J/min. Values of 12.8 (10–15.7) J/min have been reported for patients with ARDS during spontaneous breathing [16], and we found values close to these.

Although transpulmonary pressure by oesophageal pressure was not directly measured in our study, PDist_EM can be taken as an estimate of lung stress considering a standard Ecw. Therefore, the patients studied presented elevated PDist_EM with a significant difference in the APRV and BIPAP modes with respect to the PSV mode; therefore, they were at risk of lung injury associated with MV. This difference in PDist_EM can be explained by the extended inspiratory time.

Venovenous extracorporeal membrane oxygenation (VV-ECMO) in patients recovering from acute respiratory distress syndrome (ARDS) may influence spontaneous breathing for carbon dioxide removal and can be considered a lung and diaphragmatic protective strategy in patients with moderate-severe acute hypoxemic respiratory failure. In selected patients, this might be considered an alternative treatment to reduce respiratory drive and muscular effort [17, 18].

The prolonged ICU admission observed in our patients (median of 48.5 days) and poor prognosis (death 45.45%) may be the result of the development of P-SILI related more to inspiratory effort than directly to Ers; however, effort and Ers are related to each other (Fig. 1). None of the applied ventilator strategies were able to reduce inspiratory effort. COVID-19 can be considered a systemic disease, and therefore, respiratory drive, as a dependent variable, may be related to factors other than Ers that are still unclear.

Danti et al. [18] evaluated the role of sedation, PEEP, ECMO, and partial neuromuscular blockade in patients with moderate-severe hypoxemic respiratory failure as strategies to reduce inspiratory effort and provide diaphragmatic and lung protection. Inspiratory effort was evaluated using the delta of the oesophageal pressure. The study was stopped at the beginning of the COVID pandemic. We believe that our study can complement Danti´s study and provide interesting information using the Pmus signal. Overall, systematic monitoring that allows for control of muscular effort to use protective strategies to avoid diaphragmatic and lung injury during MV is important.

Experimentally, partial paralysis in severe lung injury seems to reduce P-SILi [19], and in some studies, such as Danti’s [18], it has been used to reduce elevated respiratory effort when other interventions fail. However, there is not enough evidence for clinical use, and it remains to be established.

Our study has several limitations. It represents an exploratory analysis with a limited number of patients because it is based on a case series in a monocentric design. However, we believe that this preliminary observation supports the implementation of continuous monitoring of inspiratory effort in these patients during episodes of hypoxemic ARF (AHRF) to avoid worsening respiratory function.

## Conclusions

We found high respiratory drive, effort, and WOBpin all patients studied, regardless of ventilatory mode. Monitoring inspiratory effort is essential for assessing therapeutic decisions in clinical practice. The Pmus signal is key information.

## Data Availability

The datasets used and/or analysed during the current study are available from the corresponding author upon reasonable request.

## Abbreviations

P-SILI: Patient self-inflicted lung injury
RASS: Richmond Agitation and Sedation Scale
Ers: Elastance respiratory system
Rrs: Total resistance of the respiratory system
PEEP: Positive end-expiratory pressure
PSV: Pressure support ventilation
APRV: Airway pressure release ventilation
BIPAP: BiLvel ventilation
Pmus: Muscle Pressure
Paw: Airway pressure
V´: Flow
V: Volume
P0.1: Respiratory drive
PTP_pmus: Muscle pressure time product during the inspiratory period
WOBp (Joules/min): Work of breathing of the patient
VV-ECMO: Venovenous extracorporeal membrane oxygenation
EM: Equation of motion
PDist_EM: Total distending pressure by the equation of motion
AHRF: Acute hypoxemic respiratory failure
ARDS: Acute respiratory distress syndrome
ICU: Intensive care unit
IQR: Interquartile range
FRC: Functional residual capacity
